# Manufacture of a Potential Antifungal Ingredient Using Lactic Acid Bacteria from Dry-Cured Sausages

**DOI:** 10.3390/foods12071427

**Published:** 2023-03-27

**Authors:** Tiago de Melo Nazareth, Jorge Calpe, Carlos Luz, Jordi Mañes, Giuseppe Meca

**Affiliations:** Department of Food Science and Toxicology, Faculty of Pharmacy, University of Valencia, Ave. Vicent Andrés Estellés s/n, 46100 Burjassot, Spain

**Keywords:** *Pediococcus pentosaceus*, *Lactiplantibacillus plantarum*, antifungal activity, organic acids, phenolic acids, volatile organic compounds

## Abstract

The growing interest in functional foods has fueled the hunt for novel lactic acid bacteria (LAB) found in natural sources such as fermented foods. Thus, the aims of this study were to isolate, identify, characterize, and quantify LAB’s antifungal activity and formulate an ingredient for meat product applications. The overlay method performed a logical initial screening by assessing isolated bacteria’s antifungal activity in vitro. Next, the antifungal activity of the fermented bacteria-free supernatants (BFS) was evaluated by agar diffusion assay against six toxigenic fungi. Subsequently, the antifungal activity of the most antifungal BFS was quantified using the microdilution method in 96-well microplates. The meat broth that showed higher antifungal activity was selected to elaborate on an ingredient to be applied to meat products. Finally, antifungal compounds such as organic acids, phenolic acids, and volatile organic compounds were identified in the chosen-fermented meat broth. The most promising biological candidates belonged to the *Lactiplantibacillus plantarum* and *Pediococcus pentosaceus*. *P. pentosaceus* C15 distinguished from other bacteria by the production of antifungal compounds such as nonanoic acid and phenyl ethyl alcohol, as well as the higher production of lactic and acetic acid.

## 1. Introduction

Due to their high nutritional content, meat and its derivatives are an important food category in many people’s diets. Consuming processed meats such as sausages, hot dogs, and luncheon meats has grown mainstream [1]. Between 1998 and 2018, global meat consumption rose by 58%, reaching a total of 360 million tonnes [2].

Dry-cured meat products are a type of traditional food that are manufactured and eaten across the globe. Their market dominance is well-known, owing to customers’ stringent expectations for high-quality and safe foodstuffs. Consumption of these fungus-infected foods increases the risk of exposure to mycotoxins; this is a worldwide public health concern [3].

Dry-cured meats mostly comprise muscle tissue, and their surface physical-chemical characteristics, such as low water activity (A_w_), neutrality to low pH, high salt content, and protein content, influence the microbial flora that grows on their exterior layers [4]. Although dry-fermented sausages have low A_w_ and high salt content, alterations of these properties influence the metabolism by facilitating mycotoxin biosynthesis [5].

Several species are recognized for producing mycotoxins, including *Aspergillus*, *Alternaria*, *Claviceps*, *Fusarium*, and *Penicillium* [6]. In certain environmental and substrate conditions over meat products, *Aspergillus flavus*, *A. parasiticus*, *A. ochraceus*, *P. nordicum*, *P. polonicum*, and *P. verrucosum* produce mycotoxins, posing a risk to consumers through their growth in salami, dry-cured hams, and other meat products [7,8].

*Penicillium nordicum* is the most important ochratoxin A (OTA) producing species frequently detected in cold-chain protein foods such as dry-cured ham, salami, and salted fish. In particular, this fungus grows well at temperatures of about 15 °C and salt concentrations of more than 5% NaCl [9].

The consumption of cereals contaminated with mycotoxins may result in the accumulation of these toxins in the organs of both animals and humans, thereby increasing the risk of various acute and chronic diseases. This is due to the carcinogenic, mutagenic, genotoxic, teratogenic, neurotoxic, and estrogenic effects associated with mycotoxins [10]. Mycotoxin contamination of these products may occur at any stage along the manufacturing chain, from animals contaminated in feed through the end product’s manufacture or storage. To make matters worse, despite having been detected in dry-cured meats and cheese worldwide, most nations do not regulate mycotoxins in this kind of food. Although the EU has published a new amending regulation and concluded that additional monitoring for OTA occurrence is required before setting maximum levels, this mycotoxin will soon be regulated (Commission Regulation (EC) No 2022/1370).

Various physical and chemical methods have been developed to manage the growth of fungi and their toxins. However, an effective strategy for reducing the occurrence of mycotoxins remains elusive. Furthermore, certain molds have developed resistance to chemical treatments and preservatives. Thus, reducing the prevalence of these molds in food production is of utmost importance, and significant efforts are being made to develop safe and efficient methods for this purpose. Biopreservation, which involves controlling the growth of one organism using natural substances, has garnered considerable interest in the past decade as a promising solution [11]. The quest to make healthier meat products has prompted studies to minimize saturated fat, salt, and cholesterol. Better composition of unsaturated fatty acids and integration of postbiotics, probiotics, and prebiotics were also encouraged. These functional additives may benefit human health while improving meat products’ nutrition [12]. Thus, contemporary customers desire high-quality, safe, minimally processed, and chemical-free foodstuffs.

Lactic Acid Bacteria (LAB) are either naturally present in meals or introduced as pure cultures to a variety of dietary items. LAB have a GRAS classification (generally regarded as safe), and it is estimated that fermented foods make up 25% of the European diet and 60% of the diet in many developing nations [13]. LAB are often used as starting cultures in the production of acidophilus milk, yogurt, buttermilk, cottage cheeses, hard cheeses, and soft cheeses, among other dairy products [14]. The cohabitation of LAB and yeast is also crucial for the success of other biotechnological applications, such as the production of sourdough bread [15]. Ancient traditions of employing LAB in food and animal feed, together with a new understanding of the favorable health benefits of probiotic LAB use, imply that they might serve as viable alternatives to chemical preservatives.

Regarding the antifungal activity of bioprotective cultures, it is often the consequence of the synergistic impact of many compounds since organic acids are not the only known active molecules. Antifungal action may require other molecules, such as fatty acids [16], reuterin [17], cyclic dipeptides, and proteinaceous substances [18], among others.

Phenolic acids are the most representative subgroup of phenolic compounds; they are important for fermented products because of their relationship with the sensory characteristics of foods. LAB have the ability to metabolize and release bound phenolic acids in their free form, and some of them have demonstrated a broad spectrum of antimicrobial activity, making them an important preservative to consider for foods [19]. For instance, salicylic and gallic acid have shown antifungal potential against postharvest fungi such as *P. expansum* and *F. graminearum* [20,21].

The use of probiotics in meat products is regarded as an attractive strategy for enhancing their healthfulness, as the fermentation carried out by probiotics can generate health-improving compounds, typically through the hydrolysis of polysaccharides, proteins, and fats, as well as biologically active compounds such as peptides, organic acids, and conjugated linoleic acid [22]. In addition, fermented sausages are significant matrices for probiotic delivery since they may be taken without heat treatment, which increases the survival rates of bacteria and fungi.

Therefore, a continuous survey of OTA-producing strains, especially during ripening, could support safety assurance in dry-cured meat production. Against this background, the objective of this study was to isolate LAB with antifungal properties from traditional dry-cured sausages and to develop an antifungal ingredient with potential application in the manufacture of dry-cured meat products. Additionally, the antifungal properties of the ingredient were characterized, and its responsible metabolites were identified using various analytical techniques, including high-performance liquid chromatography with diode array detection (HPLC-DAD), Quadrupole Time-of-Flight (QTOF) mass spectrometry, and Headspace Solid-Phase Microextraction (HS-SPME).

## 2. Materials and Methods

### 2.1. Chemicals

Each analyte had at least 95% of purity. Sigma-Aldrich (St. Louis, MO, USA) provided the phenolic acids 1,2–dihydroxybenzene, 3–(4–hydroxy–3–methoxyphenyl) propionic, benzoic acid, caffeic acid, gallic acid, p–Coumaric acid, sinapic acid, syringic acid, vanillic acid, and vanillin. Bachem (Bubendorf, Switzerland) provided the DL–3–phenyllactic acid (PLA). MP Biomedicals (Santa Ana, CA, USA) provided the ferulic acid. The lactic acid and acetic acid were purchased from Sigma-Aldrich (St. Louis, MO, USA).

Solvents suitable for liquid chromatography (≥99.9% purity), including acetonitrile (ACN), ethyl acetate, formic acid, and methanol, were provided by VWR Chemicals (Radnor, PA, USA). Sigma-Aldrich supplied ammonium formate (≥99.5%), C18, magnesium sulfate (MgSO_4_), sodium chloride (NaCl), and potato dextrose broth (PDB) (P6685). Liofilchem (Roseto degli Abruzzi, Teramo, Italy) provided de Man, Rogosa Sharpe broth (MRSb) (Oxoid CM359), and MRS agar (MRSa) (Oxoid CM361), plate count agar (PCA) (Oxoid CM0463) and potato dextrose agar (PDA) (Oxoid CM0139). Water was deionized with <18 MΩ/cm using a Milli-Q purification system (Millipore Corp., Bedford, MA, USA).

### 2.2. Sampling Isolation, Cultivation, and Preliminary Characterization of LAB Isolates

Sampling, isolation, cultivation, and preliminary characterization LABs were collected from various Spanish homemade-fermented products obtained from local shops in the Valencia region: “longaniza de payés”, “fuet”, “longaniza de Pascua”, and “longaniza classic”. Samples (20 g) were added to 180 mL of sterile physiological saline solution and were homogenized for 5 min. Simultaneously, bacteria were isolated by scraping a swab dipped in 0.1% peptone water (Liofilchem, Roseto degli Abruzzi, Teramo, Italy; Oxoid LP 0037) over the food surface. The swabs were then submerged in tubes containing MRSb to recover the bacteria, and the tubes were kept anaerobic at 37 °C for LAB culture. Appropriate dilutions were plated onto MRSa and cultures anaerobically for 48 h at 37 °C. Colonies were purified on MRSa after being randomly chosen from MRSa plates containing 15 to 300 colonies [23]. Microorganisms were first evaluated microscopically for Gram reaction and morphology as well as catalase production. Only Gram-positive and catalase-negative isolates were investigated, and the strains were maintained at −80 °C in MRSb containing 20% glycerol.

Biochemical and physiological tests were used to describe the microorganisms. The bacteria were evaluated for their capacity to grow at different temperatures (15, 30, and 45 °C), at different pH values (3, 5, and 6), and MRSb supplemented with NaCl (3, 6.5, and 10%).

### 2.3. Fungal Culture and Inoculum Preparation

Fungal strains purchased from the Spanish Type Culture Collection (Valencia, Spain) were *Aspergillus parasiticus* CECT 2681, *Penicillium commune* CECT 20767, *P. griseofulvum* CECT 2605–T, and *P. nordicum* CECT 2320. *A*. *flavus* ITEM 8111 was acquired from the Institute of Sciences for Food Production’s microbial culture collection (Bari, Italy). The VTT Culture Collection provided *P*. *verrucosum* VTT D–01847 (Espoo, Finlandia). The fungal strains were stored at −80 °C in sterile glycerol (30% *v*/*v*).

The fungal strains were recovered by adding 1 mL of the glycerinated solution to 9 mL PDB media. After incubating the infected PDB for 72 h at 25 °C, aliquots were plated on PDA Petri plates to harvest spores. The experiments were performed with these spores.

### 2.4. Screening of Antifungal Properties by Overlay Method

The first screening of antifungal activity was performed using bacterial isolates grown overnight in 10 mL of MRSb at 37 °C. Then, the overlay assay was used to determine the antifungal activity of LAB isolates [24]. Bacterial suspensions (10 μL) were inoculated on MRSa plates and incubated at 37 °C for 48 h. Afterward, the agar plates were filled with 10 mL of soft (7% agar) PDA containing 10^6^ conidia/mL and were incubated at 30 °C. The inhibition growth zone was determined 48 h later and measured on a cm scale. The experiments were conducted in triplicate.

### 2.5. Identification of Isolate by Matrix-Assisted Laser Desorption/Ionization Time-of-Flight Mass Spectrometry (MALDI-TOF MS) Fingerprinting

LAB was identified by extracting isolated cultures according to Maier et al. [25]. The MALDI-TOF MS was a Microflex L20 (Bruker Daltonics, Billerica, MA, USA) mass spectrophotometer equipped with an N_2_ laser. The sample spectra were acquired in positive linear ion mode, with a voltage acceleration of 20 kV, and the mass ranged from 2000 to 20,000 Da. Moreover, the spectra were obtained in triplicate as suggested by MALDI Biotyper Realtime Classification (RTC). The identification of the samples was the spectra of the largest log score organism. Finally, the results were compared with the MBT 7854 y MBT 7311_RUO database (Bruker Daltonics).

### 2.6. Preparation of the Bacteria-Free Supernatant (BFS) and Development of a Postbiotic Antifungal Ingredient

Meat broth (MB) was formulated by modifying the composition of the MRSb, as plotted in Supplementary Material Table S1. Meat extract and yeast extract were substituted with freeze-dried pork loin at different concentrations: 2.00 (MB2), 4.00 (MB4), 8.00 (MB8), and 10.00 (MB10) g/L. The ingredients were mixed and dissolved in distilled water for 10 min, and the pH was adjusted to 6. Then, the media were autoclaved at 121 °C for 21 min. Afterward, the isolated LAB were defrosted and incubated in MRSb at 37 °C for 12 h to reach the exponential growth phase. Subsequently, the LAB were placed at 10^7^ CFU/mL in 50 mL of the MB and incubated at 37 °C for 48 h. After fermentation, the LAB were mechanically separated by centrifugation at 4000× *g* for 10 min to separate the BFS. Finally, BFS were kept at −80 °C for 24 h and freeze-dried to obtain the postbiotic antifungal ingredient [26]. Freeze-dried BFS of fermented MRSb was carried out as a control group.

### 2.7. Determination of Sensitivity of Food-Borne Toxigenic Fungi to Bacteria Isolates

#### 2.7.1. In Vitro Antifungal Activity of Samples by Agar Diffusion Method

The agar diffusion test was used initially to determine the sensitivity of food spoilage and toxin-producing fungi to the BFS. The method was previously described by Fredua-Agyeman et al. [27] with some modifications.

Petri dishes containing PDA were infected with collected fungal conidial grown on PDA plates. The conidia of the fungal strains described in Section 2.3 were scraped off the agar and distributed on another PDA plate using sterile cotton swabs. Then, using sterile pipette tips, 10 mm diameter wells were created, and each well was filled with 100 μL of freeze-dried BFS resuspended in sterile water at a concentration of 250 g/L. A sterile water solution containing freeze-dried MRSb was elaborated as a control. After that, the PDA plates were incubated at 25 °C for 72 h to enable either fungal growth or BFS diffusion through the agar. Finally, the antifungal activity was determined by measuring the inhibition zone around the well on a cm scale.

#### 2.7.2. Microdilution Susceptibility Test to Freeze-Dried BFS

To establish the Minimum Inhibitory Concentration (MIC) and the Minimum Fungicidal Concentration (MFC), the microdilution test was carried out in 96-well microplates following the CLSI M38-A2 [28] criteria, with a few changes. Firstly, the microplates were filled with 100 μL of PDB. Next, plates were filled with PDB containing freeze-dried BFS at a concentration of 340 g/L and then serially diluted 2-fold to reach the final concentration of 0.33 g/L. A volume of 100 μL of PDB containing 5 × 10^3^ conidia/mL of the toxigenic fungi described by Section 2.3 was added to each well. The positive control consisted of contaminated PDB with fungal spores, whereas the negative control used PDB that had not been infected. Then, the microplates were incubated for 72 h at 25 °C. Each dose was tested in four replicates, and the experiment was carried out two times (*n* = 8).

Three parameters were determined: MIC50, MIC100, and MFC. After the incubation period, the MIC50 was established as the minimum concentration at which the BFS inhibited 50% of the fungal colonies, whereas the MIC100 was defined as the minimum concentration of BFS at which no growth was seen in the microplate. The MFC was then determined by reculturing 10 μL of the concentration matching the MIC100 and higher concentrations assessed on PDA plates. After 72 h of incubation at 25 °C, the MFC concentration that prevented fungal growth was determined. The plates were rechecked after seven days of incubation.

### 2.8. Extraction and Quantification of Organic Acids

The organic acids were extracted from the fermented MRSb and MB10, according to Özcelik et al. [29]. First, 5 mL of the BFS was aliquoted into a separated tube and treated with 1 mL of metaphosphoric acid. After 2 min of homogenization, the tubes were centrifuged at 4000× *g* for 10 min. The BFS was filtered using a membrane filter (0.45 μm). Finally, the BFS was diluted in water: formic acid (0.1%) and injected into an HPLC system.

The system applied for the chromatographic determination was an Agilent 1100 (Santa Clara, CA, USA) HPLC equipped with an autosampler, binary pump, and vacuum degasser. Results were expressed in g/L. The column used as the stationary phase was a Rezex™ ROA-Organic Acid H+ (8%) (150 × 7.8 mm, Ea Phenomenex^®^, Torrance, CA, USA). The mobile phases consisted of water as solvent A and ACN as solvent B, both acidified with 0.1% formic acid and eluted using the gradient (5% B at 0 min, 95% B at 30 min, and 5% at 35 min). Each analysis began with a 3-min equilibration of the column. The flow rate was set at 0.3 mL/min, and the sample volume was set to 20 μL. The photo diode array detector (DAD) (Agilent G1315B) was applied at 210 nm to monitor the chromatogram.

Individual stock solutions of each examined organic acid were produced by dissolving the compounds in the mobile phase. At least six-point calibration curves were created for acetic and lactic acid. The concentrations of organic acids were determined by comparing the peak area to the calibration curve standard area. Results were expressed in g/L.

### 2.9. Extraction and Quantification of Phenolic Acids

The phenolic acids of the fermented MRSb and the MB10 were purified using the QuEChERS extraction described by Brosnan et al. [30]. In 50-mL tubes, a solution of 4 g MgSO_4_, 1 g NaCl, and 10 mL ethyl acetate with 1% formic acid (*v*/*v*) were added, followed by the addition of 10 mL of the BFS. For 1 min, the tubes were vortexed and cooled on ice. Next, the solution was centrifuged at 4 °C and 4000× *g* for 10 min. The supernatant was combined with 900 mg of MgSO_4_ and 150 mg of C18 and vortexed for 1 min before centrifuging as previously described. Finally, the samples were filtered with a 0.22 μm pore filter and evaporated under nitrogen flow.

The phenolic acids analysis was performed in an Agilent 6450 Ultra High-Definition Accurate Mass QTOF-MS equipment, coupled to an Agilent Dual Jet Stream Electrospray Ionization (ESI) interface in negative ionization mode under the conditions described by Denardi-Souza et al. [31]. MassHunter Qualitative Analysis software version B.08.00 was used to handle data integration and elaboration. The results were expressed in μg/L.

### 2.10. Volatile Organic Compounds (VOCs) Determination

Volatile Organic Compounds (VOCs) emitted by the bacteria after fermentation in MRSb and MB10 media were determined by head-space solid phase microextraction (HS-SPME) technique and subsequent analysis in a gas chromatograph coupled to a mass spectrometer (GC-MS) following the methodology of Luz et al. [32] with minor modifications.

For this purpose, 10 mL of the BFS, obtained as described in Section 2.6, was placed in a 20 mL glass vial. The VOCs were extracted for 45 min at 50 °C with a divinylbenzene/carbon wide range/polydimethylsiloxane (DVB/C-WR/PDMS) coated fiber (80 µm × 10 mm) (Agilent Technologies, Santa Clara, CA, USA). Then, the fiber was desorbed for 10 min at 250 °C in splitless mode on an Agilent 7890A gas chromatograph coupled to an Agilent 7000A triple quadrupole mass spectrometer equipped with an electron impact (EI) source. The column used for chromatographic separation was an HP-5MS (30 m × 0.25 mm, 0.25 µm) (Agilent Technologies). The temperature ramp was programmed as follows: 40 °C held for 2 min and raised to 160 °C at 6 °C/min; then, it was ramped up to 260 °C at 10 °C/min and held for 4 min. The carrier gas was helium (99.99%) at a flow rate of 2.5 mL/min. Compound detection was performed in Full Scan mode in an *m*/*z* range of 40–450 Da.

The compounds were identified by comparison of their mass spectra with those recorded in the NIST 09 library. In addition, linear retention indices (LRI) were calculated based on the retention time of a solution of alkanes (C8–C20) tested under the same conditions as the samples and compared with the existing literature.

### 2.11. Statistical Analysis

For statistical analysis, GraphPad Prism version 3.0 software was employed. The differences (*p* ≤ 0.05) between the groups of the organic acids and phenolic acids composition were analyzed by a one-way ANOVA followed by the Tukey post hoc test for multiple comparisons.

## 3. Results and Discussion

### 3.1. Isolation, Screening, and Identification of Antifungal Bacteria Strains

**Figure 1:**
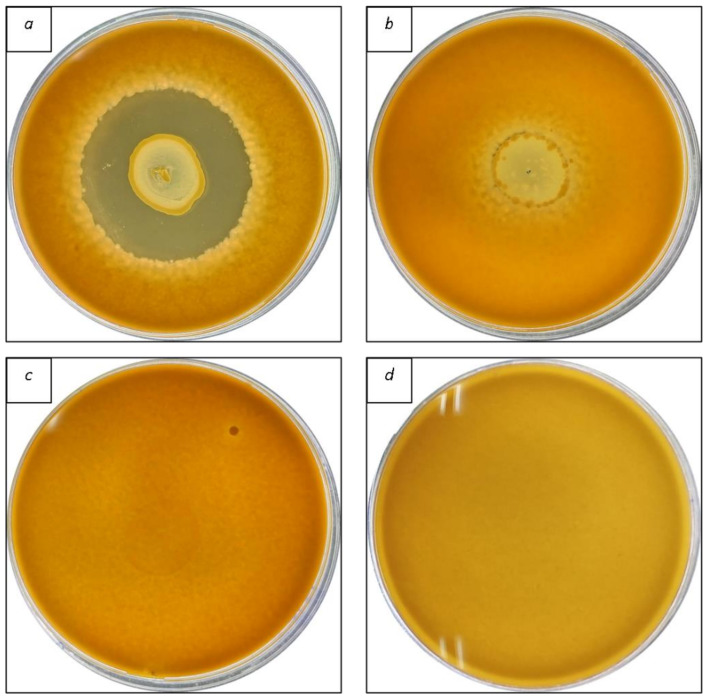
Screening of lactic acid bacteria (LAB) through overlay method. Strong inhibitory action (**a**); slightly inhibitory effect (**b**); no inhibitory action (**c**); control (**d**).

A total of 102 bacteria were isolated from different handmade dry Spanish sausages. Among these, 42 isolates were classified as LAB since they were gram-positive, could grow in micro-aerobic conditions, and lacked catalase activity (Table 1). Further characterization of bacteria strains highlighted that these isolates grew in the following conditions: temperature ranging from 15 to 45 °C; pH ranging from 3.5 to 6; and growth in MRS-NaCl at 3, 6.5, and 10%. Therefore, these bacteria were selected, and after the characterization, a first screening was performed using the overlay approach to assess the antifungal properties of the 42 bacteria isolated; they were assayed against six spoilage fungal strains in vitro that commonly contaminate cured sausage products. As presented in Table 2, only 14 bacteria isolates (C11, C12, C13, C15, C20, C28, C56, C58, C60, C66, C69, C71, C72, and C79) demonstrated inhibitory activity against all fungi examined, with varying degrees of inhibition depending on the fungal strain tested. In particular, *P. griseofulvum* CECT 2605–T and *P. commune* CECT 20767 were the most susceptible strains, with inhibition zones ranging from 1.5–3.0 cm and 1.0–3.0 cm, respectively. In contrast, *Aspergillus* strains (*A. parasiticus* CECT 2681 and *A. flavus* ITEM 8111) were the most resistant to the LAB, with inhibition zones ranging from 0.3–1.7 cm. An example of overlay methodology is given in Figure 1. These findings agree with those obtained by previous authors [33], which noticed that *Aspergillus* strains are more resistant to LAB than other fungal genera.

The 14 gram-positive bacteria with antifungal activity against all fungal strains were identified by MALDI-TOF-MS and classified regarding the MTB 7854 and MBT 7311_RUO databases (Bruker Daltonics). The classification to species level was the following: *Pediococcus pentosaceus* C11, *P. pentosaceus* C12, *P. pentosaceus* C13, *P. pentosaceus* C15, *Lactiplantibacillus plantarum* C20, *P. pentosaceus* C28, *P. pentosaceus* C56, *P. pentosaceus* C58, *L. plantarum* C60, *P. pentosaceus* C66, *P. pentosaceus* C69, *P. pentosaceus* C71, *P. pentosaceus* C72, and *P. pentosaceus* C79. Thus, the bacteria identified with potential inhibitory properties were studied to develop an antifungal ingredient against dry-cured meat spoilage agents.

The LAB with antifungal activity are poorly documented, while more attention has been exploited its antibacterial activity. The LAB that produces bacteriocins are isolated from a severe category of food. For example, Delcarlo et al. [34] isolated 22 LAB with antimicrobial action from mussels on the Argentina coast. Parlindungan et al. [35] identified novel probiotic candidates from fermented meats and characterized them by bacteriocin production. Furthermore, Ivanovic et al. [36] isolated, identified, and characterized antibacterial LAB from traditional cheese.

Only a few studies from scientific literature cite a broad spectrum of antifungal activity for LAB. Most of them showed higher strain-specific antifungal activity against one or two mold species [37]. For this reason, one of the goals of this study is to isolate a large-spectrum strain. In this work, we observed that antifungal activity was strain-dependent, as well as fungal species and methodologies analyzed. Moreover, it seems that dry-cured sausages can be used as a potential source of antifungal LAB because they have significant antagonistic properties against the microorganisms tested.

### 3.2. In Vitro Antifungal Study of the MB Formulated

Two methods were performed to determine the fungal sensitivity to bacterial isolates: agar diffusion test and microdilution of BFS in 96-well plates.

The MB was formulated by changing the composition of the MRSb by subtracting the yeast extract and meat extract and adding freeze-dried pork loin as a protein source at 2.00, 4.00, 8.00, and 10.00 g/L (MB2, MB4, MB8, and MB10, respectively). These new formulations were fermented with the 14 bacteria strains identified in Section 3.1, and a first qualitative evaluation was performed using the agar-diffusion test to assess the antifungal properties. Moreover, the fermented MRSb was also prepared with the same bacteria strains to compare the effectiveness of the newly formulated ingredient.

After fermentation, only the MB10 exhibited antifungal activity similar to the fermented MRSb used as a control reference. As presented in Table 3, the inhibition halos obtained varied according to the fungal strain tested and the bacteria used as inoculum. In particular, the most sensitive strains to the MB10 were *P. commune* CECT 20767, *P. nordicum* CECT 2320, and *P. verrucosum* VTT–D01847. These strains were susceptible to all the formulated MB, with inhibition halos observed on the PDA plates ranging from 0.2 cm (+) to inhibition halos greater than 0.4 cm (+++). The fungal strains with the most significant resistance were those of the *Aspergillus* genera, in which only *Pediococcus pentosaceus* C12, *Pediococcus pentosaceus* C15, and *Lactiplantibacillus plantarum* C60 showed inhibitory activity. This finding agrees with the previous characterization of the LAB isolates by overlay method since *Aspergillus* strains showed higher resistance to the LAB than *Penicillium* strains. For this reason, the formulation MB10, *P. pentosaceus* C12, *P. pentosaceus* C15, and *L. plantarum* C60 isolates were selected, and the preliminary results of the antifungal activity were confirmed through the MIC50, MIC100, and MFC determination in vitro. The MB10 and MRSb that were fermented for 48 h obtained the highest inhibition halo zone. The antifungal effect of MB10 fermented for 24, 48, and 72 h is plotted in Table S2.

Similar findings in the literature indicated that a range of theories might explain LAB’s antifungal activities; Schnürer and Magnusson [38] found that the suppression of mold growth on an agar plate is the consequence of a complex interaction of multiple chemicals and metabolites, all of which contribute to the overall antifungal activity.

The microdilution assay demonstrated that the BFS of the fermented MRSb was the most active extract against all fungi tested, regardless of the bacteria isolated (Table 4). The MIC50 obtained ranged from 5–21 g/L; the MIC100 ranged from 21–85 g/L; and the MFC ranged from 21–170 g/L. However, for MB10 formulation, the MIC50, MIC100, and MFC parameters ranged from 11–43 g/L, 21–85 g/L, and 43–170 g/L, respectively. It is important to underline that the BFS of the MRSb and the MB10 fermented by *P. pentosaceus* C15 showed lower concentrations required to achieve fungal growth against all the toxigenic strains tested compared to *P. pentosaceus* C12 and *L. plantarum* C60 fermented BFS. Although the BFS of the MRSb evidenced a higher antifungal effect, a poor difference was evidenced when compared to MB10. In some cases, there were no significant differences (*p* ≤ 0.05) between the treatments, e.g., MB10 fermented by *P. pentosaceus* C15 had no statistical difference to MRSb comparing the average values of MIC100 and MFC (Table 4). In other words, *P. pentosaceus* C15 was able to ferment MB10 and produce antifungal compounds, which inhibited fungal growth at similar conditions to MRSb. In addition, MRSb fermented by *P. pentosaceus* C15 obtained MIC50, MIC100, and MFC values lower than *L. plantarum* C60 and *P. pentosaceus* C12, regardless of the fungal strain essayed.

The MRSb was applied in both experiments as a control group since previous studies have demonstrated its antifungal activity while being fermented by LAB and producing antimicrobial substances. For instance, Taroub et al. [39] identified *P. pentosaceus* and *L. plantarum* strains that showed good antifungal activity against *A. niger* and *A. carbonarius*. In addition, the strains showed a high capacity for the degradation of OTA, one of the main toxins produced by these fungi. However, the authors could not identify the compounds responsible for the antifungal and antimicotoxigenic activities of the strains. In other studies, the application of MRS fermented by *L. plantarum* inhibited the *A. flavus and F. verticillioides* growth and reduced the production of aflatoxin and fumonisin in cereals such as maize [26].

Therefore, MRSb has proven to be an interesting medium for fermenting bacteria as antimicrobial metabolites are produced. However, the use of MRSb has some disadvantages, such as its high cost. In addition, the complexity of some of their ingredients that convert MRSb might not be allowed as an ingredient in meat products. Therefore, one of the objectives of the work was to develop a meat broth with similar antifungal characteristics but, at the same time, could be incorporated as an antifungal ingredient.

Overall, the *P. pentosaceus* C15 showed higher antifungal potential in the agar diffusion test and microdilution of BFS; hence, these results led us to select this strain for the fermentation of MB10 and elaborate a postbiotic antifungal ingredient.

### 3.3. Chemical Characterization of the Postbiotic Antifungal Ingredient

#### 3.3.1. Organic and Phenolic Acid Production

**Figure 2:**
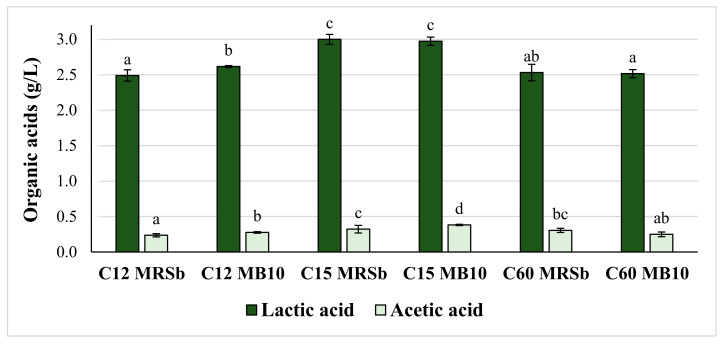
The concentration of organic acids produced by LAB in meat broth (MB10) and MRS broth (MRSb) after incubation for 48 h at 37 °C. The meat broth was prepared with 10% freeze-dried loin pork and fermented by *P. pentosaceus* C12, *P. pentosaceus* C15, and *L. plantarum* C60. Different letters represent statistical differences in the same group of molecules between treatments (*p* ≤ 0.05).

The metabolites generated after fermentation of the antifungal strains selected were investigated in the BFS of the MRSb and MB10. As plotted in Figure 2, two organic acids, lactic acid and acetic acid, were identified in both formulations. For lactic acid, the mean values of this metabolite ranged from 2.49 to 3.00 g/L, whereas for acetic acid, the concentration detected was lower, with mean values ranging from 0.24 to 0.38 g/L. The highest amount of lactic acid was detected in the fermented MRSb (2.973 g/L) and MB10 (3.00 g/L) fermented by the *P. pentosaceus* C15 strain. There were no statistical differences in lactic acid production between these two formulations (*p* ≤ 0.05). Regarding acetic acid, the higher concentration (*p* ≤ 0.05) was also quantified in the fermented MB10 with *P. pentosaceus* C15 (0.38 g/L), followed by the MRSb of *P. pentosaceus* C15 (0.32 g/L) and the fermented MRSb with *L. plantarum* C60 (0.31 g/L).

Concerning the phenolic acids, a total of 12 different metabolites, described in the literature as antifungals, were identified and quantified through the UHPLC Q-TOF MS technique. Among them, it is noteworthy that the concentration of benzoic acid, DL–3–phenyllactic acid, syringic acid, and vanillic acid stood out regardless of the fermented broth and bacteria tested (Table 5). In general, it could be noted that MRSb produced higher concentrations of phenolic acids compared to its analog MB10. However, in most cases the difference was not significant (*p ≤* 0.05). In view of these results, we suggest further studies to evaluate the synergistic effect of these four compounds in order to evaluate their antifungal activity and to increase the understanding about the mechanism of antimicrobial action of LAB.

The selected microorganisms grew on the MB10 and produced a broad variety of metabolites previously described in the literature as antimicrobial compounds. The phenolic acid most produced by *P. pentosaceus* C12 in MB10 was syringic acid, showing values of 19.47 µg/L: these values are significantly higher than bacteria C60. Aziz et al. [40] demonstrated that syringic acid at 300 mg/L inhibited *A. flavus and A. parasiticus* growth and aflatoxin production. In addition, Ren et al. [41] demonstrated that lower doses of syringic acid (100 mg/L) avoided the growth of *A*. *niger*. Therefore, it seems that a higher concentration of phenolic acids is required to achieve an antifungal effect when they are applied in isolation. These findings reinforce the hypothesis that the LAB antifungal effect is obtained via a synergistic effect.

Regarding *P. pentosaceus* C15 and *L*. *plantarum* C60, vanillic acid was the most produced phenolic acid, reaching values of 25.18 and 24.00 µg/L in MB10 and 30.83 and 27.60 µg/L in MRSb, respectively. An oxidized derivative of vanillin, vanillic acid, is a monohydroxybenzoic acid composed of a 4–hydroxybenzoic acid with a methoxy group at position 3 [41]. It has been noted for its antioxidant properties, but its antifungal activity is poorly reported, and more research is required. In contrast, its precursor, vanillin, has been the subject of extensive research, and its antifungal activity varies depending on the microorganism tested [42,43].

These findings also suggest that the antifungal ingredient obtained from MB10 could be incorporated into meat food products to provide a significant source of phenolic and organic acids.

LAB can synthesize a wide variety of antifungal compounds such as organic acids, phenolic acids, antimicrobial peptides, diacetyl, and reuterin [44]. During fermentation, carbon metabolism produces organic acids such as lactic acid, acetic acid, and propionic acid [45]. Among these metabolites, the most studied are organic acids. It is important to mention that these metabolites can constitute synergistic activity. However, this synergistic activity’s exact mechanism is unknown [46].

The results also suggest that the combination of organic acids and phenolic acids produced by LAB could be responsible for the inhibitory activity of the spoilage fungi in vitro. However, it is essential to underline that the antifungal properties of the MB10 are probably not exclusively produced by organic and phenolic acids; for instance, several volatile substances can act synergistically and potentialize the antifungal properties [16]. Moreover, the decrease in pH collaborates to a more efficient antifungal activity [47]. In this context, Peyer et al. [48] found that organic acids and phenolic acids generated by some LAB strains as antifungal metabolites have synergistic actions against *F. culmorum*. Likewise, in bakery products, organic acids and antifungal peptides produced by LAB showed a significant synergistic effect which allowed biopreservation and enhanced the shelf life of quinoa and rice bread [49].

In addition to the synergistic compound effect, the combination of different microorganisms can also promote a positive effect. For instance, Ruggirello et al. [50] associated yeast with LAB and obtained a great antifungal effect against strains of *Aspergillus* and *Penicillium* genera, fermenting cocoa beans. The authors reported that the antifungal potential could be a result of association between organic acids of LAB with proteinaceous substances of yeasts. According to Christ-Ribeiro et al. [51], phenolic chemicals avoid fungal development by inhibiting the production of cell wall components including glucan, chitin, and mannoproteins, as well as cell membrane components like ergosterol. This process happens by damaging the cell wall and cell membrane, affecting nutrient influx regulation. As a result, phenolic chemicals impede fungal cell production of proteins, amino acids, and sphingolipids. Moreover, they obstruct electron transit and the preservation of cellular integrity.

#### 3.3.2. VOC Analysis in MB10 Formulation

The fermented meat broth VOCs (MB10) were analyzed through HS-SPME coupled to the GC-MS technique. A total of 24 compounds were identified through MS comparison with the NIST library and calculation of the LRI and belonged to several chemical classes: acid (2), alcohol (8), aldehyde (6), ketone (5), and pyrazine (2) (Table S3). Moreover, the percentage peak area (%PA) of each identified compound was calculated, and the results obtained are reported in Table 6.

**Figure 3:**
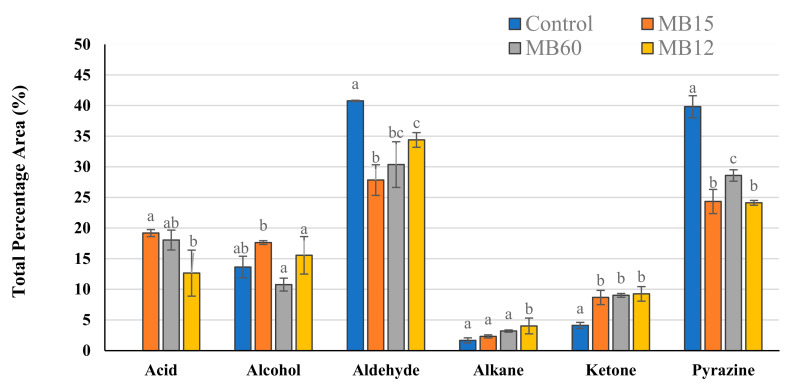
Total percentage area (%) of the chemical classes identified in the volatile fraction of the Meat Broth formulated with 10% of lyophilized pork loin and fermented by *P. pentosaceus* C12 (MB12), *P. pentosaceus* C15 (MB15), and *L. plantarum* C60 (MB60). Different letters represent statistical differences in the same group of molecules between treatments (*p* ≤ 0.05).

Aldehydes were the most abundant compounds in the samples analyzed and represented a proportion between 27.8–40.8% of the total VOCs detected (Figure 3). In particular, five linear aldehydes (heptanal, octanal, nonanal, 2–decenal, and dodecanal) were identified in all MB10; nonetheless, the greatest observed concentration was of an aromatic aldehyde, benzeneacetaldehyde, which %PA ranged between 18.4 and 20.3% depending on the formulation analyzed. Saturated aldehydes are lipid-derived volatiles produced mainly by the oxidation of oleic acid, a characteristic fatty acid of raw pork meat [52,53,54]. Regarding benzeneacetaldehyde, this compound could be synthesized using phenylalanine as a precursor through the Maillard reaction during the sterilization step of the MB10 since it is mainly detected in cooked meat [55,56]. It was noted that aldehyde content in the fermented MB10 was statistically lower (*p* ≤ 0.05) in comparison with the control group, which corroborated the findings of Kwaw et al. [57], which described a decline of aldehydes in mulberry juice when LAB fermentation was applied.

Pyrazines were the second abundant group detected in MB10 formulations (%PA ranging from 24.1 to 39.8). It was observed that the %PA statistically decreased in the fermented MB10 formulations (*p* ≤ 0.05) compared to control formulations. This phenomenon was also described by Kurt et al. [58], which evidenced that the pyrazine content in spirulina water solutions (4% *w*/*v*) was reduced after LAB fermentation.

The greatest variety of chemical compounds found in the MB10 were alcohols, representing a mean %PA value ranging from 10.8 to 17.6%. Among the alcohols identified, phenylethyl alcohol (PEA) was detected in a higher proportion (6.6 ± 0.5 %PA) in the MB10 fermented with C15 (MB10-C15) in comparison with other formulations (*p* ≤ 0.05). This active compound has been studied for its antifungal potential and could explain the lower MIC and MFC values detected in the MB10-C15, as previously reported in Section 3.2. For instance, Gong et al. [59] determined that the antifungal properties of *Enterobacter absuriae* Vt–7 were mainly due to the volatile antifungal PEA, and it effectively controlled the development of the toxigenic fungi *Aspergillus flavus* in peanuts. Similar results were obtained by Wonglom et al. [60], who associated the antifungal potential of *Trichoderma* sp. T76–12/2 against *Sclerotium* fruit rot due to the synthesis of PEA and other VOCs.

Regarding ketones, LAB not only significantly increased (*p* ≤ 0.05) the ketone levels in the formulated MB10, but also introduced three new ketones that were not present in the control group, such as 2–heptanone, 2–undecanone, and 2–tridecanone (Table 6). Some ketones may be synthesized through microbial oxidation of fatty acids, and this could explain its higher proportion in the MB10 formulations when compared to the control formulation (MB10 without fermentation) [61].

Only two acids, acetic acid and nonanoic acid, were identified in the fermented MB10. Nonanoic acid is mainly produced from the degradation of unsaturated fatty acids such as oleic acid, whereas acetic acid is produced because of the heterofermentative metabolism of LAB [62]. Previous studies conducted observed an increase in these chemical compounds when LAB are employed in different food matrices such as pumpkin and watermelon juices [63,64]. Regarding its biological properties, it is important to emphasize that nonanoic acid has evidenced antimicrobial properties against several pathogens such as *Alternaria alternata*, *Botrytis cinerea* [65], *Candida albicans* [66], *Salmonella enterica* [67], and *Escherichia coli* O157:H7 [68]. Thus, volatile acids combined with the other biological compounds found in the MB10 (such as PEA, organic acids, and phenolic acids) could contribute to the antifungal properties of the formulated ingredient since LAB antifungal potential is related to the synergistic action of the different metabolites synthesized [38].

#### 3.3.3. Principal Component Analysis (PCA) of the MB10 with LAB

**Figure 4:**
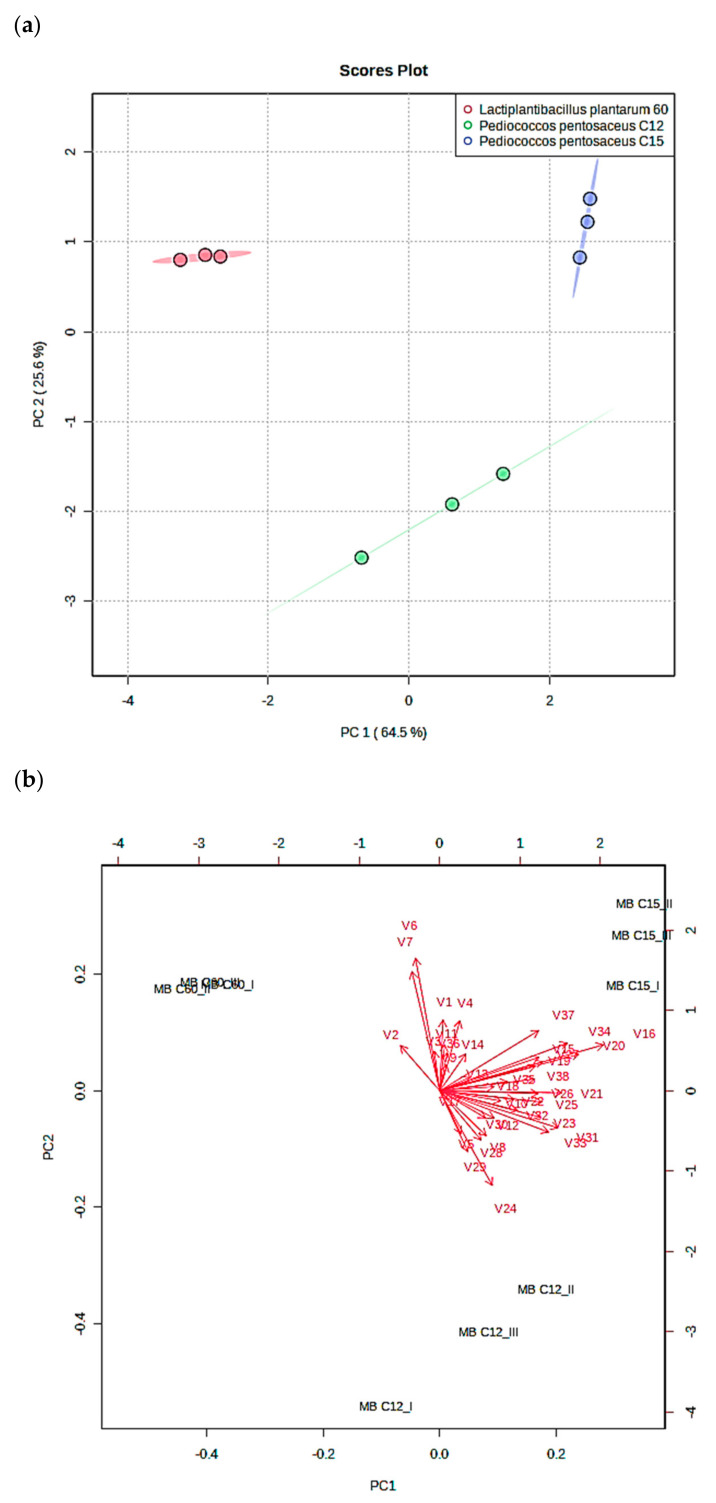
Principal component analysis (PCA) scores plot of the bioactive compounds (organic acids, phenolic acids, and volatile organic compounds) found in the meat broth fermented by *P. pentosaceus* C12 (MB C12), *P. pentosaceus* C15 (MB C15), and *L. plantarum* C60 (MB C60) (**a**) and relative loadings of the variables employed (**b**).

In order to understand the differences between bioactive metabolite production (organic acids, phenolic acids, and VOCs) in the fermented MB formulations, a PCA was realized, and the results obtained are outlined in Figure 4. The sum of the first two principal components (PC) achieved 90.1% of the total explained variance, in which PC1 explained up to 64.5% of the total variance, while PC2 explained 25.6% (Figure 4a). The PC1 distributed on the positive axis both *P. pentosaceus* strains (C12 and C15), whereas *L. plantarum* C60 strain was positioned on the negative axis. The PC2 allowed the distinction of the C12 and C15 strains according to their bioactive metabolite profile since C12 was positioned on the negative axis and C15 was positioned on the positive axis.

According to the loading plot that represents the relative importance of the variables analyzed (Figure 4b), MB10-C12 was distinguished from MB10-C15 for its higher volatile aldehyde production; specifically, this was found for specific compounds such as heptanal (V24), 2–decenal (V28), and dodecanal (V29). The MB10 formulated with C15 was mainly characteristic from the other formulations due to the higher production of different antifungal compounds such as nonanoic acid (V16) and phenyl ethyl alcohol (V20).

Regarding the MB10 formulated with *L. plantarum* strain (MB10-C60), the main variable that distinguishes this formulation from those prepared with *P. pentosaceus* strains was the higher production of the phenolic compound 3–(4–hydrodoxy–3–methoxyphenyl) propionic (V2). Furthermore, the ferulic acid (V6) and gallic acid content (V7) positioned on the positive axis according to the second component the MB10-C60 formulation and permitted the distinction between the MBC12 formulation. The variable that was positioned on the negative axis according to the first component of the MB C60 was the lower content in volatile alcohols in comparison with C12 and C15, such as 1–nonanol (V21) and 2–undecanol (V23).

## 4. Conclusions

In the study, 42 antifungal LAB isolated from dry-cured sausages were evaluated against 6 fungi of the *Aspergillus* and *Penicillium* genera. Firstly, a battery of bacteria was isolated from dry-cured meat products and characterized according to their antifungal properties. Among them, 14 presented antifungal capacity and 3 inhibited the growth of all fungi assayed. The *P. pentosaceus* C15 showed higher antifungal activity in vitro; these bacteria were selected for the preparation of several fermented-meat broth (MB2, MB4, MB8 and MB10). MB were elaborated changing de MRSb formulation. The MB10 and MB formulated with 10% of freeze-dried loin pork demonstrated higher antifungal activity through agar diffusion and microdilution method.

In particular, the MB10 fermented with *P. pentosaceus* C15 was selected to elaborate a postbiotic antifungal ingredient due to their higher antifungal activity. The chemical characterization highlighted that the MB10 formulation was rich in phenolic acids and organic acids such as lactic acid and acetic acid. Moreover, the postbiotic product changed the VOCs composition and increased the concentration and number of antifungal compounds detected.

Discovering the synergistic process amongst antifungal agents might provide understanding in increasing the effect while modifying the implicated bacterial or nutritional compositions, subsequently leading to food applications. PCA analysis indicated that some antifungal agents enabled us to differentiate strains by their production. For instance, *P. pentosaceus* C15 and C12 were distinguished by the production of aldehydes such as heptanal, decenal, and dodecanal. In contrast, *L. plantarum* C60 was differentiated from *P. pentosaceus* strains due to the production of 3–(4–hydrodoxy–3–methoxyphenyl) propionic.

Further research will focus on the application of the postbiotic antifungal ingredient (MB10-C15) in the development of dry-cured meat products, as well as study c inhibition of mycotoxins synthesis that can potentially harm the consumers health.

## Figures and Tables

**Table 1:** Characterization of LAB isolated from dry-cured sausages.

Preliminary Characterization				Growth at Temperature	Growth in pH	Growth in MRS-NaCl (%)	
Bacteria	Morphology	Gram *	Catalase	15 to 45 °C	3.5 to 6	3 to 6.5	10
C1	Coccoid	P	-	+	+	+	-
C4	Coccoid	P	-	+	+	+	-
C5	Coccoid	P	-	+	+	+	-
C6	Coccoid rod	P	-	+	+	+	-
C11	Coccoid	P	-	+	+	+	-
C12	Coccoid	P	-	+	+	+	-
C13	Coccoid	P	-	+	+	+	-
C14	Coccoid rod	P	-	+	+	+	+
C15	Coccoid	P	-	+	+	+	+
C16	Rod-shaped	P	-	+	+	+	+
C17	Rod-shaped	P	-	+	+	+	+
C19	Coccoid	P	-	+	+	+	+
C20	Rod-shaped	P	-	+	+	+	+
C27	Rod-shaped	P	-	+	+	+	+
C28	Coccoid	P	-	+	+	+	+
C29	Rod-shaped	P	-	+	+	+	+
C30	Rod-shaped	P	-	+	+	+	+
C31	Coccoid	P	-	+	+	+	+
C35	Coccoid	P	-	+	+	+	-
C36	Coccoid	P	-	+	+	+	+
C37	Coccoid	P	-	+	+	+	+
C38	Coccoid	P	-	+	+	+	-
C39	Coccoid	P	-	+	+	+	+
C44	Coccoid	P	-	+	+	+	+
C45	Coccoid	P	-	+	+	+	+
C46	Coccoid rod	P	-	+	+	+	-
C47	Coccoid	P	-	+	+	+	+
C48	Coccoid	P	-	+	+	+	+
C50	Coccoid	P	-	+	+	+	+
C56	Coccoid	P	-	+	+	+	+
C57	Coccoid	P	-	+	+	+	+
C58	Coccoid	P	-	+	+	+	+
C59	Coccoid	P	-	+	+	+	-
C60	Rod-shaped	P	-	+	+	+	+
C66	Coccoid	P	-	+	+	+	+
C68	Coccoid	P	-	+	+	+	+
C69	Coccoid	P	-	+	+	+	+
C70	Coccoid	P	-	+	+	+	-
C71	Coccoid	P	-	+	+	+	+
C72	Coccoid	P	-	+	+	+	+
C79	Coccoid	P	-	+	+	+	+
C80	Coccoid	P	-	+	+	+	+

* P means positive; + signifies visual growth, and - means no visual growth.

**Table 2:** Antifungal activity of LAB by overlay technique.

Selected Bacteria	*Aspergillus parasiticus*	*Aspergillus flavus*	*Penicillium commune*	*Penicillium verrucosum*	*Penicillium griseofulvum*	*Penicillium nordicum*
	Inhibition Zone (cm)					
C1	0.0	0.0	2.4	2.0	3.0	1.0
C4	0.0	0.1	1.5	1.7	3.0	0.9
C5	0.3	0.0	2.5	1.4	3.0	1.0
C6	0.3	0.0	2.7	1.7	3.0	1.1
C11	1.0	0.4	3.0	2.0	3.0	1.4
C12	0.5	0.3	3.0	1.7	2.3	1.3
C13	0.7	0.6	3.0	1.8	2.1	1.0
C14	0.5	0.0	2.0	1.4	3.0	1.1
C15	0.4	0.6	3.0	1.6	3.0	1.2
C16	0.5	0.0	3.0	1.5	0.6	0.7
C17	0.3	0.0	0.6	2.0	3.0	0.8
C19	0.3	0.0	1.6	1.7	3.0	1.0
C20	0.8	0.9	3.0	1.7	1.9	1.3
C27	0.5	0.0	0.9	1.7	1.3	0.5
C28	1.0	0.8	3.0	1.7	1.8	1.3
C29	0.4	0.0	2.9	1.7	1.2	0.9
C30	0.6	0.0	3.0	1.4	1.0	0.9
C31	0.5	0.0	3.0	1.7	1.8	0.9
C35	0.4	0.0	3.0	1.7	1.6	1.1
C36	0.0	0.0	2.7	1.6	3.0	1.0
C37	0.4	0.0	3.0	2.5	1.0	0.8
C38	0.2	0.0	2.4	1.5	3.0	1.1
C39	0.4	0.0	0.0	1.8	1.6	0.4
C44	2.0	0.0	1.4	1.7	3.0	1.0
C45	0.4	0.0	1.1	1.6	3.0	0.8
C46	0.2	0.0	0.5	2.0	3.0	0.7
C47	0.3	0.0	3.0	1.6	2.5	1.1
C48	0.5	0.0	3.0	1.7	1.5	1.1
C50	0.9	0.0	2.5	1.5	1.8	0.8
C56	1.5	1.2	3.0	2.1	1.6	1.1
C57	1.0	0.0	2.8	1.8	0.2	0.6
C58	1.5	1.4	3.0	1.5	3.0	1.5
C59	0.5	0.0	0.6	2.0	1.5	0.5
C60	1.2	1.7	1.6	1.5	2.5	1.2
C66	1.0	1.1	1.0	2.1	0.8	0.6
C68	1.0	0.0	3.0	2.2	2.5	1.1
C69	0.4	0.5	3.0	2.5	3.0	1.1
C70	0.0	0.0	3.0	1.3	3.0	1.2
C71	1.5	1.2	2.6	1.5	3.0	1.3
C72	1.0	1.1	3.0	1.5	1.5	0.9
C79	0.4	0.3	2.7	1.7	3.0	1.1
C80	0.0	0.0	2.7	1.7	3.0	1.0

**Table 3:** Antifungal activity of bacterial free supernatant (BFS) extract in agar diffusion method. LAB fermented MRS broth (MRSb) and a meat broth (MB10) for 48 h at 37 °C. Then, the supernatant was freeze-dried and resuspended at a concentration of 250 g/L. These bacteria were previously selected due to their antifungal effect in the overlay method.

Selected Bacteria	Antifungal Activity of the BFS of the Fermented MRS Broth and the Meat Broth (MB10)											
	Fungal Strain											
	*A. flavus*		*A. parasiticus*		*P. commune*		*P. griseofulvum*		*P. nordicum*		*P. verrucosum*	
	MRSb	MB10	MRSb	MB10	MRSb	MB10	MRSb	MB10	MRSb	MB10	MRSb	MB10
C11	+	-	-	-	+	+	+	+	+	+	+	+
C12	+	+	++	+	++	++	++	++	+	+	++	++
C13	-	-	+	-	+	+	+	+	+++	+++	+++	+++
C15	++	++	++	++	+++	+++	+++	+++	+++	+++	+++	+++
C20	-	-	+	-	+	+	+	+	++	++	+	+
C28	+	-	-	-	+	+	+	+	+++	+++	+++	+++
C56	-	-	+	-	+	+	+	+	+	+	+	+
C58	-	-	-	-	+	+	++	++	+++	+++	+++	+++
C60	+	+	++	++	++	++	++	++	+++	+++	++	++
C66	+	-	-	-	++	++	+++	+++	++	++	+++	+++
C69	-	-	+	-	+	+	-	-	++	++	+	+
C71	-	-	+	-	+	+	-	-	+	+	+	+
C72	+	-	-	-	++	+	++	++	++	++	+++	+++
C79	+	-	-	-	++	+	++	++	++	++	+++	+++

(-) Represents no halo inhibition; (+) Represents a growth inhibition halo of 0.2 cm; (++) represents a growth inhibition halo of between 0.2 to 0.4 cm; (+++) represents a growth inhibition halo greater than 0.4 cm.

**Table 4:** Minimal inhibitory concentration (MIC) and minimal fungicidal concentration (MFC) of bacterial-free supernatant (BFS). MRS broth (MRSb) and meat broth (MB10) were fermented by LAB for 48 h at 37 °C and centrifuged to obtain the BFS.

Fungi	BFS Concentration (g/L) of the Fermented MRSb								
	*Lactiplantibacillus plantarum* C60			*Pediococcus pentosaceus* C12			*Pediococcus pentosaceus* C15		
	MIC ^1^	MIC ^2^	MFC	MIC ^1^	MIC ^2^	MFC	MIC ^1^	MIC ^2^	MFC
*A. flavus*	11	85	170	21	85	170	11	21	85
*A. parasiticus*	11	43	170	11	43	170	11	21	85
*P. commune*	21	43	85	21	43	85	11	21	85
*P. griseofulvum*	21	43	170	21	43	170	5	21	170
*P. nordicum*	11	43	85	11	43	85	5	21	21
*P. verrucosum*	21	43	85	21	43	85	11	43	43
Mean	16	50	128	18	50	128	9	25	82
Fungi	BFS concentration (g/L) of the MB10								
	MIC ^1^	MIC ^2^	MFC	MIC ^1^	MIC ^2^	MFC	MIC ^1^	MIC ^2^	MFC
*A. flavus*	21	85	170	43	85	170	21	43	85
*A. parasiticus*	21	43	170	21	43	170	21	43	85
*P. commune*	43	85	85	43	85	85	21	21	85
*P. griseofulvum*	43	85	170	43	85	170	11	21	170
*P. nordicum*	21	43	85	43	85	85	11	21	43
*P. verrucosum*	43	85	85	43	85	85	11	43	43
Mean	32 *	71	128	39 *	78 *	128	16 *	35	85

^1^: 50% of visual inhibition growth; ^2^: 100% of visual inhibition growth. *: results were statistically analyzed by the *t*-test, comparing the Bacterial free supernatant (BFS) of MRSb with the BFS of MB10. Means are significantly different if *p* ≤ 0.05.

**Table 5:** The concentration of phenolic compounds in Man, Rogosa and Sharpe broth (MRSb) and meat broth (MB10) fermented by antifungal LAB. The broths were fermented for 48 h at 37 °C.

Phenolic Compound	The Concentration of Phenolic CompoundsMean ± SD (μg/L)					
	*Pediococcus pentosaceus* C12		*Pediococcus pentosaceus* C15		*Lactiplantibacillus plantarum* C60	
	MRSb	MB10	MRSb	MB10	MRSb	MB10
1,2–Dihydroxybenzene	9.39 ± 2.02 ^a^	2.51 ± 0.41 ^b^	5.86 ± 2.74 ^cd^	5.64 ± 2.22 ^cd^	7.12 ± 1.3 ^d^	4.64 ± 1.39 ^c^
Propionic acid	7.14 ± 1.79 ^a^	7.08 ± 1.34 ^a^	9.66 ± 2.64 ^a^	9.36 ± 0.62 ^a^	6.19 ± 2.85 ^a^	13.44 ± 5.58 ^b^
Benzoic acid	31.65 ± 4.42 ^a^	18.09 ± 5.35 ^b^	24.69 ± 0.72 ^c^	22.12 ± 7.32 ^bc^	19.38 ± 5.91 ^bc^	23.18 ± 0.35 ^bc^
Caffeic acid	4.07 ± 1.46 ^a^	3.99 ± 2.46 ^a^	8.37 ± 1.26 ^b^	7.72 ± 1.87 ^bc^	3.40 ± 0.97 ^a^	6.27 ± 1.55 ^c^
DL–3–Phenyllactic acid	18.17 ± 3.52 ^a^	18.30 ± 1.54 ^ab^	31.90 ± 7.49 ^c^	22.68 ± 3.25 ^b^	16.98 ± 3.66 ^a^	15.85 ± 0.61 ^a^
Ferulic acid	n.d	n.d	4.79 ± 1.81 ^ab^	5.85 ± 4.02 ^ab^	3.70 ± 0.72 ^a^	6.18 ± 1.26 ^b^
Gallic acid	n.d	n.d	8.04 ± 2.83 ^a^	6.06 ± 1.88 ^a^	6.58 ± 2.05 ^a^	7.44 ± 1.25 ^a^
p–Coumaric acid	22.59 ± 9.91 ^a^	9.37 ± 1.72 ^b^	6.28 ± 0.07 ^b^	7.56 ± 0.44 ^b^	5.29 ± 1.47 ^b^	4.99 ± 2.03 ^b^
Sinapic acid	4.87 ± 1.04 ^ab^	6.43 ± 0.44 ^ac^	5.85 ± 2.38 ^ad^	7.86 ± 3.66 ^cd^	3.54 ± 0.86 ^b^	7.22 ± 1.32 ^cd^
Syringic acid	26.78 ± 3.52 ^a^	19.47 ± 5.28 ^b^	17.73 ± 4.12 ^b^	21.12 ± 0.39 ^b^	17.42 ± 1.40 ^b^	12.83 ± 3.40 ^c^
Vanillic acid	20.84 ± 4.56 ^ac^	18.81 ± 2.82 ^a^	30.83 ± 6.19 ^b^	25.18 ± 1.13 ^d^	27.60 ± 2.94 ^bd^	24.00 ± 2.12 ^cd^
Vanillin	11.68 ± 1.26 ^a^	13.76 ± 1.08 ^a^	16.85 ± 5.47 ^a^	13.73 ± 1.72 ^a^	7.57 ± 1.65 ^b^	8.46 ± 1.51 ^b^

Different letters mean a significant statistical difference in the concentration of phenolic compounds among fermented broths (*p ≤* 0.05). n.d means not detected.

**Table 6:** Percentage area (%) of the volatile organic compounds (VOCs) identified in the formulated meat broth (MB10) fermented with different lactic acid bacteria strains (*P. pentosaceus* C12, *P. pentosaceus* C15, and *L. plantarum* C60). Results are expressed as mean ± standard deviation.

N°	Rt	Compound	Control	C15	C60	C12
Acid						
1	3.11	Acetic acid	n.d	13.74 ± 1.12 ^a^	11.11 ± 0.62 ^ab^	8.95 ± 2.63 ^b^
2	15.49	Nonanoic acid	n.d	5.45 ± 1.69 ^a^	6.94 ± 1.00 ^a^	3.70 ± 1.13 ^a^
Alcohol						
3	8.97	2–Octanol	8.12 ± 2.16 ^a^	1.77 ± 0.16 ^b^	0.39 ± 0.05 ^b^	0.87 ± 0.28 ^b^
4	11.19	1–Octanol	2.16 ± 0.07 ^a^	2.70 ± 0.10 ^ab^	3.03 ± 0.25 ^ab^	4.16 ± 1.11 ^b^
5	11.62	2–Nonanol	n.d	0.58 ± 0.08 ^a^	0.53 ± 0.14 ^a^	0.66 ± 0.06 ^a^
6	13.43	Phenylethyl alcohol	1.24 ± 0.13 ^a^	6.57 ± 0.45 ^b^	1.60 ± 0.20 ^a^	2.44 ± 0.88 ^a^
7	13.6	1–Nonanol	1.26 ± 0.17 ^a^	3.65 ± 0.05 ^b^	1.95 ± 0.18 ^ab^	3.80 ± 1.49 ^b^
8	15.88	1–Decanol	n.d	1.22 ± 0.12 ^a^	2.18 ± 0.70 ^a^	1.86 ± 0.35 ^a^
9	16.27	2–Undecanol	0.86 ± 0.03 ^a^	1.15 ± 0.16 ^a^	1.09 ± 0.03 ^a^	1.76 ± 0.42 ^b^
Aldehyde						
10	6.25	Heptanal	3.51 ± 0.43 ^ab^	1.04 ± 0.21 ^b^	2.13 ± 0.35 ^b^	5.71 ± 2.66 ^a^
11	8.89	Octanal	13.45 ± 2.80 ^a^	3.81 ± 0.33 ^b^	3.75 ± 0.63 ^b^	4.98 ± 0.06 ^b^
12	11.02	Benzeneacetaldehyde	20.31 ± 2.76 ^a^	19.27 ± 2.39 ^a^	19.82 ± 3.34 ^a^	18.36 ± 3.74 ^a^
13	11.42	Nonanal	1.24 ± 0.13 ^a^	2.91 ± 0.53 ^a^	2.43 ± 0.16 ^a^	2.94 ± 1.44 ^a^
14	15.82	2–Decenal	0.97 ± 0.18 ^a^	0.53 ± 0.07 ^a^	1.44 ± 0.13 ^a^	1.47 ± 0.72 ^a^
15	18.5	Dodecanal	1.28 ± 0.07 ^a^	0.27 ± 0.05 ^b^	0.81 ± 0.19 ^ab^	0.94 ± 0.56 ^ab^
Alkane						
16	10.77	Decane, 2–methyl	n.d	0.85 ± 0.05 ^a^	2.42 ± 0.04 ^b^	1.81 ± 0.63 ^b^
17	11.69	Undecane	1.66 ± 0.41 ^ab^	1.47 ± 0.31 ^ab^	0.78 ± 0.22 ^a^	2.21 ± 0.66 ^b^
Ketone						
18	5.78	2–Heptanone	n.d	1.73 ± 0.40 ^a^	2.80 ± 0.53 ^a^	2.82 ± 0.72 ^a^
19	8.71	2–Octanone	2.52 ± 0.45 ^a^	2.89 ± 0.19 ^a^	1.92 ± 0.15 ^a^	2.32 ± 0.95 ^a^
20	11.28	2–Nonanone	1.59 ± 0.03 ^ab^	2.82 ± 0.55 ^b^	0.88 ± 0.05 ^a^	2.13 ± 1.28 ^ab^
21	16.11	2–Undecanone	n.d	0.63 ± 0.10 ^a^	1.29 ± 0.38 ^b^	0.82 ± 0.12 ^ab^
22	20.45	2–Tridecanone	n.d	0.60 ± 0.08 ^a^	2.13 ± 0.35 ^b^	1.13 ± 0.20 ^a^
Pyrazine						
23	4.55	Pyrazine, methyl–	10.24 ± 1.00 ^a^	5.30 ± 0.09 ^b^	6.91 ± 1.73 ^b^	4.34 ± 1.11 ^b^
24	6.37	Pyrazine, 2,5–dimethyl	29.58 ± 2.79 ^a^	19.05 ± 1.87 ^b^	21.68 ± 0.80 ^b^	19.79 ± 0.07 ^b^

n.d = no-detected. Different letters represent statistical differences in the same molecules between treatments (*p* ≤ 0.05).

## Data Availability

The data presented in this study are available on request from the corresponding author.

## Supplementary Materials

The following supporting information can be downloaded at: https://www.mdpi.com/article/10.3390/foods12071427/s1, Table S1: Elaboration of meat broths for fermentation for lactic acid bacteria; Table S2: Antifungal activity of formulated meat broths (MB) and MRS broth fermented by Pediococcus pentosaceus C15 during 24, 48, and 72 h at 37 °C. The bacterial-free supernatant (BFS) was freeze-dried, resuspended at a concentration of 500 g/L, and tested against six toxigenic fungi; Table S3: Identification of Volatile Organic Compounds (VOCs) of the fermented Meat Broth 10, with retention time, chemical class, calculated LRI (LRI exp.), and references [69,70,71,72,73,74,75,76,77,78,79,80,81,82,83,84,85,86].
