# Supplementary material for: Manufacture of a Potential Antifungal Ingredient Using Lactic Acid Bacteria from Dry-Cured Sausages
Source: Foods. 2023 Mar 27;12(7):1427. doi: 10.3390/foods12071427 (PMC10093346; doi:10.3390/foods12071427)
Supplement: Supplementary file 1 [file foods-12-01427-s001.zip › foods-2269158-supplementary.pdf]

Article

Manufacture of a potential antifungal ingredient using lactic acid bacteria from dry-cured sausages

Tiago de Melo Nazareth [1,*], Jorge Calpe [1], Carlos Luz [1], Jordi Mañes [1], Giuseppe Meca [1]

[1] Department of Food Science and Toxicology, Faculty of Pharmacy, University of Valencia, Ave. Vicent Andrés Estellés s/n, 46100 Burjassot, Spain
* Correspondence: tiago@uv.es; Tel.: +34963544959

Table S1. Elaboration of meat broths for fermentation for lactic acid bacteria.

**Ingredient**	*Concentration (g/L)*				
	**MB2**	**MB4**	**MB8**	**MB10**	**MRSb**
Dextrose	20.00	20.00	20.00	20.00	20.00
Meat extract	-	-	-	-	8.00
Yeast extract	-	-	-	-	4.00
Peptone	10.00	10.00	10.00	10.00	10.00
Sodium acetate	5.00	5.00	5.00	5.00	5.00
Dipotassium phosphate	2.00	2.00	2.00	2.00	2.00
Ammonium citrate	2.00	2.00	2.00	2.00	2.00
Magnesium sulfate	0.20	0.20	0.20	0.20	0.20
Manganese sulfate	0.05	0.05	0.05	0.05	0.05
Tween (mL/L)	1.00	1.00	1.00	1.00	1.00
Lyophilized pork loin	2.00	4.00	8.00	10.00	-

Table S2. Antifungal activity of formulated meat broths (MB) and MRS broth fermented by *Pediococcus pentosaceus* C15 during 24, 48, and 72 h at 37 °C. The bacterial-free supernatant (BFS) was freeze-dried, resuspended at a concentration of 500 g/L, and tested against six toxigenic fungi.

**Fungal strain**	**BFS of *Pediococcus pentosaceus* C15 (500 g/L)**					
	**24 h**		**48 h**		**72 h**	
	**MRSb**	**MB10**	**MRSb**	**MB10**	**MRSb**	**MB10**
Aspergillus flavus	+	-	++	++	+	+
Aspergillus parasiticus	+	-	++	+	+	+
Penicillium commune	++	+	+++	+++	+++	++
Penicillium griseofulvum	++		+++	+++	++	++
Penicillium nordicum	++	+	+++	+++	++	++
Penicillium verrucosum	++	+	+++	+++	+++	++

(+) Represents a growth inhibition halo of 0.2 cm; (++) represents a growth inhibition halo of between 0.2 to 0.4 cm; (+++) represents a growth inhibition halo greater than 0.4 cm.

Table S3. Identification of Volatile Organic Compounds (VOCs) of the fermented Meat Broth 10, with retention time, chemical class, calculated LRI (LRI exp.), and references.

Nº	Rt	Compound	Class	Identif.	LRI exp.	LRI lit.	Reference
1	3.11	Acetic acid	Acid	MS			
2	4.55	Pyrazine, methyl-	Pyrazine	MS + LRI	805	807	[1]
3	5.78	2-Heptanone	Ketone	MS + LRI	859	859	[2]
4	6.25	Heptanal	Aldehyde	MS + LRI	880	882	[3]
5	6.37	Pyrazine, 2,5-dimethyl	Pyrazine	MS + LRI	885	883	[4]
6	8.71	2-Octanone	Ketone	MS + LRI	980	984	[5]
7	8.89	Octanal	Aldehyde	MS + LRI	988	991	[6]
8	8.97	2-Octanol	Alcohol	MS + LRI	991	990	[7]
9	10.77	Decane, 2-methyl	Alkane	MS + LRI	1063	1061	[8]
10	11.02	Benzeneacetaldehyde	Aldehyde	MS + LRI	1073	1071	[9]
11	11.19	1-Octanol	Alcohol	MS + LRI	1080	1079	[10]
12	11.28	2-Nonanone	Ketone	MS + LRI	1083	1083	[11]
13	11.42	Nonanal	Aldehyde	MS + LRI	1089	1089	[4]
14	11.62	2-Nonanol	Alcohol	MS + LRI	1097	1098	[12]
15	11.69	Undecane	Alkane	MS + Std	1100		
16	13.43	Phenylethyl alcohol	Alcohol	MS	1172		
17	13.60	1-Nonanol	Alcohol	MS + LRI	1180	1180	[13]
18	15.49	Nonanoic acid	Acid	MS + LRI	1261	1263	[3]
19	15.82	2-Decenal	Aldehyde	MS + LRI	1275	1270	[14]
20	15.88	1-Decanol	Alcohol	MS + LRI	1278	1279	[15]
21	16.11	2-Undecanone	Ketone	MS + LRI	1289	1273	[3]
22	16.27	2-Undecanol	Alcohol	MS + LRI	1296	1294	[16]
23	18.50	Dodecanal	Aldehyde	MS + LRI	1398	1405	[17]
24	20.45	2-Tridecanone	Ketone	MS + LRI	1493	1497	[18]

References:

1. Lu, C.-Y.; Hao, Z.; Payne, R.; Ho, C.-T., Effects of water content on volatile generation and peptide degradation in the Maillard reaction of glycine, diglycine, and triglycine, **J. Agric. Food Chem.**, 2005, 53, 16, 6443-6447, https://doi.org/10.1021/jf050534p
2. Pino, J.A.; Marbot, R.; Bello, A., Volatile compounds of *Psidium salutare* (H.B.K.) Berg. fruit, **J. Agric. Food Chem.**, 2002, 50, 18, 5146-5148, https://doi.org/10.1021/jf0116303
3. Ziegenbein, F.C.; Hanssen, H.-P.; König, W.A., Secondary metabolites from *Ganoderma lucidum* and *Spongiporus leucomallellus*, **Phytochemistry**, 2006, 67, 2, 202-211, https://doi.org/10.1016/j.phytochem.2005.10.025
4. Pino, J.; Almora, K.; Marbot, R., Volatile components of papaya (*Carica papaya* L., maradol variety) fruit, **Flavour Fragr. J.**, 2003, 18, 6, 492-496, https://doi.org/10.1002/ffj.1248
5. Sampaio, T.S.; Nogueira, P.C.L., Volatile components of mangaba fruit (*Hancornia speciosa* Gomes) at three stages of maturity, **Food Chem.**, 2006, 95, 4, 606-610, https://doi.org/10.1016/j.foodchem.2005.01.038
6. Xian Q.; Chen H.; Zou H.; Yin D., Chemical composition of essential oils of two submerged macrophytes, *Ceratophyllum demersum* L. and *Vallisneria spiralis* L., **Flavour Fragr. J.**, 2006, 21, 3, 524-526, https://doi.org/10.1002/ffj.1588
7. Nickavar B.; Kamalinejad M.; Mohandesi S., Comparison of the components of the essential oils from leaves and fruits of *Grammosciadium platycarpum*, **Chem. Nat. Compd.**, 2006, 42, 6, 686-688, https://doi.org/10.1007/s10600-006-0252-x
8. Pavlovic, M.; Tzakou, O.; Petrakis, P.V.; Couladis, M., The essential oil of *Hypericum perforatum* L., *Hypericum tetrapterum* Fries and *Hypericum olympicum* L. growing in Greece, **Flavour Fragr. J.**, 2006, 21, 1, 84-87, https://doi.org/10.1002/ffj.1521
9. Bredie, W.L.P.; Mottram, D.S.; Guy, R.C.E., Effect of temperature and pH on the generation of flavor volatiles in extrusion cooking of wheat flour**, J. Agric. Food Chem.**, 2002, 50, 5, 1118-1125, https://doi.org/10.1021/jf0111662
10. Bylaite, E.; Meyer, A.S., · Characterisation of volatile aroma compounds of orange juices by three dynamic and static headspace gas chromatography techniques, **Eur. Food Res. Technol.**, 2006, 222, 1-2, 176-184, https://doi.org/10.1007/s00217-005-0141-8
11. Cardeal, Z.L.; da Silva, M.D.R.G.; Marriott, P.J., Comprehensive two-dimensional gas chromatography/mass spectrometric analysis of pepper volatiles, **Rapid Commun. Mass Spectrom.**, 2006, 20, 19, 2823-2836, https://doi.org/10.1002/rcm.2665
12. Demetzos, C.; Angelopoulou, D.; Perdetzoglou, D., A comparative study of the essential oils of *Cistus salviifolius* in several populations of Crete (Greece), **Biochem. Syst. Ecol.**, 2002, 30, 7, 651-665, https://doi.org/10.1016/S0305-1978(01)00145-4
13. Gocmen, D.; Gurbuz, O.; Rouseff, R.L.; Smoot, J.M.; Dagdelen, A.F., Gas chromatographic-olfactometric characterization of aroma active compounds in sun-dried and vacuum-dried tarhana, **Eur. Food Res. Technol.**, 2004, 218, 6, 573-578, https://doi.org/10.1007/s00217-004-0913-6
14. Zhao J.Y.; Liu J.M.; Zhang X.Y.; Liu Z.J.; Tsering T.; Zhong Y.; Nan P., Chemical composition of the volatiles of three wild *Bergenia* species from western China, **Flavour Fragr. J.**, 2006, 21, 3, 431-434, https://doi.org/10.1002/ffj.1689
15. Mahattanatawee, K.; Goodner, K.L.; Baldwin, E.A., Volatile constituents and character impact compounds of selected Florida's tropical fruit, **Proc. Fla. State Hort. Soc.**, 2005, 118, 414-418
16. Viana, F.A.; Andrade-Neto, M.; Pouliquen, Y.B.M.; Lucien, V.G., Chemical composition of the essential oil from roots of *Philodendron acutatum* Schott., **J. Essent. Oil Res.**, 2002, 14, 3, 172-174, https://doi.org/10.1080/10412905.2002.9699814

17. Kukic J.; Petrovic S.; Pavlovic M.; Couladis M.; Tzakou O.; Niketic M., Composition of essential oil of *Stachys alpina* L. ssp *dinarica* Murb., **Flavour Fragr. J.**, 2006, 21, 3, 539-542, https://doi.org/10.1002/ffj.1684
18. Benkaci-Ali, F.; Baaliouamer, A.; Meklati, B.Y.; Chemat, F., Chemical composition of seed essential oils from Algerian *Nigella sativa* extracted by microwave and hydrodistillation, **Flavour Fragr. J.**, 2007, 22, 2, 148-153, https://doi.org/10.1002/ffj.1773
